# Design for strong absorption in a nanowire array tandem solar cell

**DOI:** 10.1038/srep32349

**Published:** 2016-08-30

**Authors:** Yang Chen, Mats-Erik Pistol, Nicklas Anttu

**Affiliations:** 1 Division of Solid State Physics and NanoLund, Lund University, Box 118, 22100 Lund, Sweden

## Abstract

Semiconductor nanowires are a promising candidate for next-generation solar cells. However, the optical response of nanowires is, due to diffraction effects, complicated to optimize. Here, we optimize through optical modeling the absorption in a dual-junction nanowire-array solar cell in terms of the Shockley-Quessier detailed balance efficiency limit. We identify efficiency maxima that originate from resonant absorption of photons through the HE11 and the HE12 waveguide modes in the top cell. An efficiency limit above 40% is reached in the band gap optimized Al_0.10_Ga_0.90_As/In_0.34_Ga_0.66_As system when we allow for different diameter for the top and the bottom nanowire subcell. However, for experiments, equal diameter for the top and the bottom cell might be easier to realize. In this case, we find in our modeling a modest 1–2% drop in the efficiency limit. In the Ga_0.51_In_0.49_P/InP system, an efficiency limit of η = 37.3% could be reached. These efficiencies, which include reflection losses and sub-optimal absorption, are well above the 31.0% limit of a perfectly-absorbing, idealized single-junction bulk cell, and close to the 42.0% limit of the idealized dual-junction bulk cell. Our results offer guidance in the choice of materials and dimensions for nanowires with potential for high efficiency tandem solar cells.

The use of III-V nanowires for p-i-n junction solar cells is an emerging avenue for photovoltaics^1^,^2^,^3^,^4^,^5^,^6^. Both single wire^4^,^6^,^7^ and large-area nanowire array^5^,^8^,^9^ devices show promise for next generation solar cells. Already for single nanowire systems, diffraction of light can lead to resonant coupling of light into the nanowire with several absorption peaks as a function of wavelength^10^,^11^. Optimization of the geometry of the single-nanowire geometry is necessary to obtain maximum photocurrent^12^ and open circuit voltage^4^,^12^. The resonances can lead to a 20 times stronger absorption per volume semiconductor material in a III-V nanowire as compared to a bulk sample^13^.

An array of nanowires gives in turn access to large-area devices when higher output power is needed. For such arrays, an efficiency of 13.8% has been demonstrated using InP nanowires with a single p-i-n junction in the axial direction^5^, and an efficiency of 15.3% has been reached with GaAs nanowires^9^. However, the use of a single material gives an upper limit for the amount of sun light that can be converted into electrical energy^14^, due to two reasons. First, the energy of photons with energy below the band gap energy of the semiconductor cannot be utilized since those low-energy photons cannot be absorbed. Second, a large part of the energy of absorbed high-energy photons is wasted due to thermalization. In this thermalization process, the photogenerated electrons and holes relax in energy to their respective band edges.

To reach higher efficiencies in solar cells, an avenue is to use multiple semiconductors, epitaxially grown on top of each other^15^. See Fig. 1 for a system with two different semiconductor materials, where one material is used in the top cell and a different material in the bottom cell. The idea in such a tandem device is to absorb high energy photons in a high band gap top cell. In that top cell, the thermalization loss of the high energy photons is decreased compared to the single junction cell. The lower energy photons continue to the bottom cell where they are absorbed. Due to the lower band gap of the bottom cell than in the single junction cell, more photons are absorbed than in the single junction cell. In this way, the tandem cell can absorb more photons than the single junction cell, while at the same time having reduced thermalization losses. However, in planar cells, the crystal lattice constant between materials in adjacent subcells/layers should be matched to yield high-quality materials without performance limiting dislocations. Such requirements on crystal-lattice matching limit strongly the choice of materials for tandem cells.

Nanowire structures offer a clear benefit for multi-junction solar cells compared with planar cells. Efficient strain relaxation in nanowires allows for the fabrication and combination of dislocation-free, highly lattice-mismatched materials^16^,^17^,^18^,^19^. Furthermore, III-V semiconductor nanowire arrays can in principle be fabricated on top of a Si substrate^19^, giving the prospect of using the Si substrate as the bottom cell^2^,^20^,^21^,^22^,^23^.

Thus, nanowires offer freedom for the material choice in multi-junction solar cells, making it easy to reach optimum material combinations to match the solar spectrum. Furthermore, the resonant absorption by designing the nanowire geometry holds the prospect of lower material usage than in thin-films^13^. Therefore, to enable high-efficiency nanowire tandem solar cells, we need to understand the optimum choice of materials for the subcells as well as the optimum nanowire geometry to have the best absorption characteristics for photovoltaics. Already for single junction nanowire-array cells, we know that both the array pitch and the nanowire diameter need to be optimized simultaneously. At the same time, the optimum diameter depends on the band gap of the solar cell, that is, on the material choice^8^.

Here, we perform optical modeling to calculate and optimize the absorption of light in a dual junction tandem nanowire solar cell (Fig. 1) with the scattering matrix method^1^,^8^,^13^,^24^,^25^. This modeling allows us to perform a Shockley-Queisser detailed balance analysis to study and optimize the efficiency potential of the nanowire solar cell as a function of material choice and geometrical design of the nanowires. We show that an efficiency limit above 40% can be reached in the band gap optimized Al_0.10_Ga_0.90_As/In_0.34_Ga_0.66_As system when we allow for different diameter *D*_top_ and *D*_bot_ for the top and the bottom subcell. However, for experiments, the case of *D*_top_ = *D*_bot_ might be easier to realize. In this case, we find a 1–2% drop in the efficiency. In the experimentally relevant Ga_0.51_In_0.49_P/InP system, an efficiency limit of *η* = 37.3% is reached for a nanowire length of 13 μm when using equal diameters of *D*_top_ = *D*_bot_ = 160 nm and a pitch *P* = 380 nm (we analyze also the effect of varying nanowire length, with results summarized in Table 1). These efficiencies for nanowire tandem cells are well above the 31.0% limit of an idealized, perfectly absorbing single-junction bulk cell and close to the 42.0% limit of the idealized, band gap optimized dual-junction bulk cell.

## Material choice for the top and the bottom cell in a nanowire tandem solar cell

To choose the materials for the top and the bottom nanowire subcell, we perform the well-known Shockley-Queisser detailed balance analysis^14^ assuming first perfect absorption of above band gap photons in each subcell^22^,^26^. This analysis corresponds to the case when each subcell absorbs optimally, without reflection losses. The specific assumption and technical details of the analysis can be found in the Supplementary Information.

Importantly, we assume that cell 1 absorbs all photons of energy above *E*_1_, the band gap energy of cell 1. Cell 2 absorbs in turn all photons with energies between *E*_1_ and *E*_2_, the band gap energy of cell 2. We assume that *A*_1_(*λ*) = 1 for *λ* < *λ*_1,bg_ and *A*_1_(*λ*) = 0 otherwise. Here, *A*_1_(*λ*) [*A*_2_(*λ*)] is the absorption spectrum of cell 1 (2), that is, the fraction of incident light of wavelength *λ* absorbed in cell 1 (2). Similarly, we assume that *A*_2_(*λ*) = 1 for λ_1,bg_ < λ < λ_2,bg_ and *A*_2_(*λ*) = 0 otherwise. Here, *λ*_1,bg_ = 2πћc/*E*_1_ and *λ*_2,bg_ = 2πћc/*E*_2_. In this way, we find the materials that maximize the efficiency limit of the nanowire solar cell when the geometry is designed for optimum absorption (Fig. 2). Note that below, in the section *Geometry Design*, when considering the effect of the nanowire geometry on the absorption, we model the absorption spectra *A*_1_(*λ*) ≤ 1 and *A*_2_(*λ*) ≤ 1 for each choice of the geometry, which includes sub-optimal absorption and varying reflection losses.

We find a maximum efficiency of 42.0% when the band gaps of the top and the bottom cell are *E*_1_ = 1.58 eV and *E*_2_ = 0.95 eV respectively (Fig. 2). Note that the results in Fig. 2 are in good agreement with previous detailed-balance calculations of multi-junction bulk cells^22^,^26^. In our case, for the modeling of the emission to the substrate, we use a refractive index of *n* = 3.5 to represent the InP substrate. We note that the emission of photons into this high-refractive index substrate has caused a 3% decrease in this maximum efficiency.

To choose the III-V materials for the nanowire subcells, we calculated first the band gap for varying ternary compounds^27^. After this, we investigated which ternary compounds have tabulated, experimentally determined, reliable refractive index values available for the optics modelling. Among the ternaries for which such refractive index data were readily available, we identified Al_0.10_Ga_0.90_As (band gap of 1.55 eV^27^, and refractive index from ref. [28]) for the top cell and In_0.34_Ga_0.66_As (band gap of 0.95 eV^27^, and refractive index from ref. [29]) for the bottom cell as a good material combination with an efficiency limit of 40.7%.

However, we could imagine that the fabrication of a dual-junction nanowire solar cell could benefit from the knowledge and control of the fabrication of single-junction nanowire solar cells. In this case, the natural candidates are the well-performing InP^5^ and GaAs^9^. The band gaps of both these materials work well for the bottom cell (see inset in Fig. 2 for an InP bottom cell and Supplementary Figure S1 for a GaAs bottom cell). However, the surface recombination velocity of unpassivated GaAs can be five orders of magnitude higher than that of unpassivated InP^30^. Therefore, GaAs nanowires need dedicated surface passivation schemes,^9^ whereas the requirement on surface passivation is relieved for InP nanowires^5^. Therefore, we chose to concentrate on an InP bottom cell. Here, a maximum efficiency of 38.6% is found with a top cell band gap energy of 1.86 eV (inset of Fig. 2). We note that Ga_0.51_In_0.49_P, for which refractive index data is available^31^, has a band gap energy of 1.85 eV^31^, giving an efficiency limit of 38.5% in the tandem configuration with InP. Depending on the surface properties of the GaInP, this GaInP/InP system could perhaps even provide the prospect of high efficiency without dedicated surface passivation schemes. Thus, we study the efficiency limit of both the AlGaAs/InGaAs and the GaInP/InP system.

## Geometry Design

**Figure 1:**
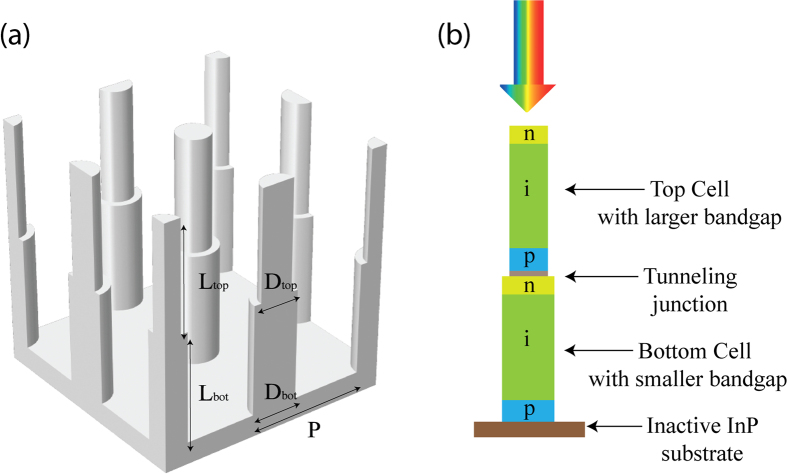
(**a**) Schematic diagram and geometry parameters of a dual junction nanowire array on an inactive substrate. (**b**) Schematic of a possible realization of the electrical design with axially configured p-i-n junction subcells with a tunnel junction to connect the top and the bottom subcell.

**Figure 2:**
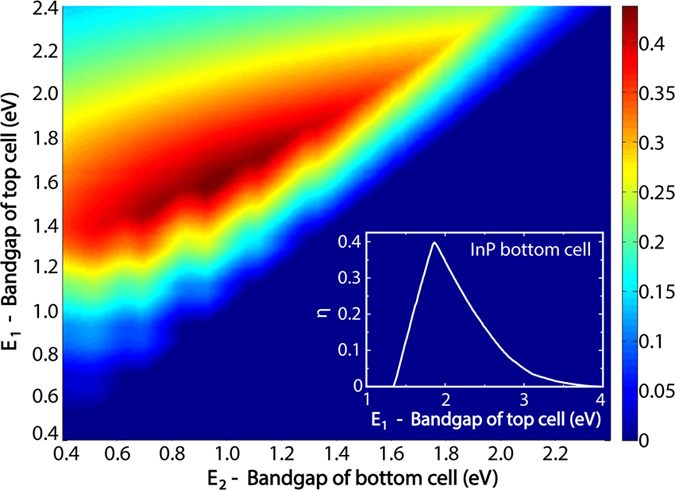
Shockley-Queisser detailed balance efficiency as a function of material band gaps for perfectly absorbing subcells. The maximum efficiency *η* = 42.0% shows up at *E*_1_ = 1.58 eV and *E*_2_ = 0.95 eV for the top and the bottom cell band gap, respectively. Notice that in this analysis for the perfectly absorbing subcells, for *E*_1_ < *E*_2_ the bottom cell (cell 2) does not absorb any photons. Thus, *j*_2_ = 0 and consecutively the current through this current-matched series-connected solar cell is zero, leading to *η* = 0. The inset shows the efficiency limit for varying top cell band gap for the case of an InP bottom cell, that is, when *E*_2_ = 1.34 eV.

After choosing the materials for the top and the bottom cell as described above (the nearly band gap optimized Al_0.10_Ga_0.90_As/In_0.34_Ga_0.66_As system as well as the technologically relevant Ga_0.51_In_0.49_P/InP system), we turn to consider the geometry of the nanowire subcells (Fig. 1). There are five geometry parameters: the length of each subcell (*L*_top_ and *L*_bot_), the diameter of each subcell (*D*_top_ and *D*_bot_), and the pitch (*P*) of the square array, which need to be optimized with respect to the absorption (*A*_1_(*λ*) and *A*_2_(*λ*)) of light in each subcell.

Different computational methods, such as the finite-element method (FEM)^3^,^32^,^33^, the rigorous coupled wave analysis (RCWA)^4^,^6^ and the scattering matrix method^1^,^8^,^13^,^24^,^25^, have been used for studying the diffraction and absorption of light in nanostructures through the solution of the Maxwell equations, which give results in good agreement with experiments^13^. We chose to employ the scattering matrix method to solve the Maxwell equations for normally incident light in order to calculate the absorption spectrum *A*_1(2)_(λ) of the nanowire top and bottom cells. We use tabulated refractive index values *n*(*λ*) for the Al_0.10_Ga_0.90_As^28^, In_0.34_Ga_0.66_As^29^, Ga_0.51_In_0.49_P^31^, and InP^34^. We then calculate the Shockley-Queisser detailed balance efficiency (see Supplementary Information Eqs. (S1)–(S6) for technical details).

Note that the optics modeling is done with the nanowires on top of an InP substrate (see Fig. 1). However, absorption of light in the substrate does not contribute to the current or voltage of the solar cell in our analysis. Thus, the substrate functions optically merely to partially reflect the light that reaches the substrate. Therefore, a change to a different substrate, like the less-expensive Si^19^, with similar *n* ≈ 3.5 as the InP would give very similar absorption spectra.

We start by considering the case of Al_0.10_Ga_0.90_As (band gap of *E*_1_ = 1.55 eV) for the top cell and In_0.34_Ga_0.66_As (band gap of *E*_2_ = 0.95 eV) for the bottom cell, which was found to be a good band gap combination with efficiency limit of 40.7% for perfectly absorbing subcells.

**Figure 3:**
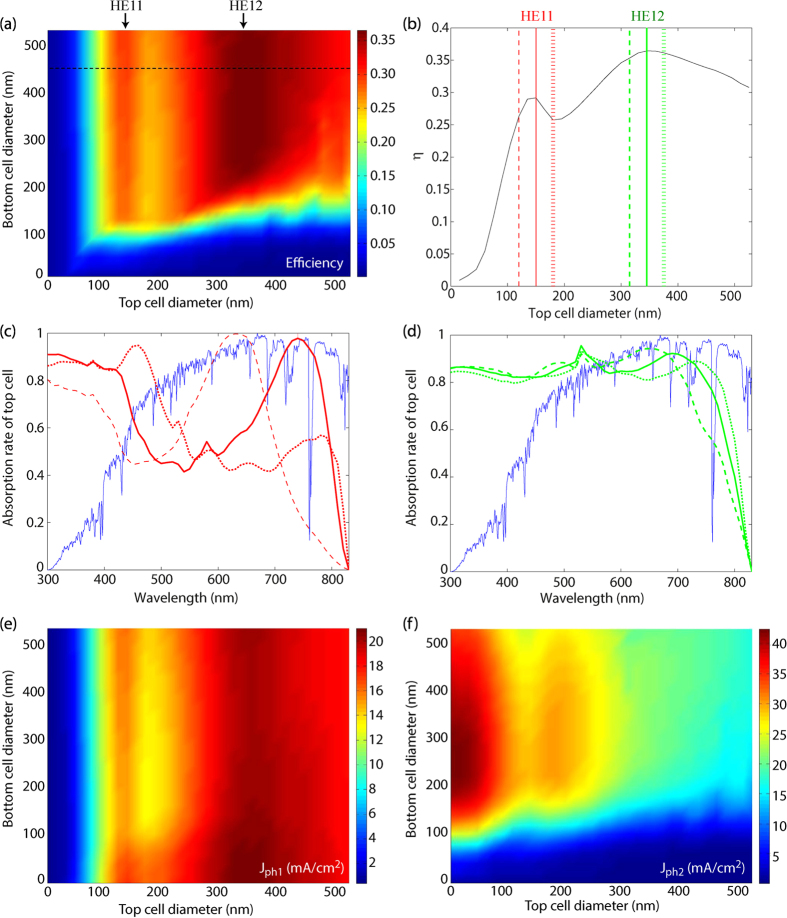
(**a**) Efficiency limit as a function of *D*_top_ and *D*_bot_ for *P* = 530 nm, *L*_top_ = 2000 nm, and *L*_bot_ = 2900 nm. Here, the top cell is of Al_0.10_Ga_0.9_As and the bottom cell of In_0.34_Ga_0.66_As. (**b**) Efficiency limit as a function of top cell diameter as extracted from the dashed black line in (**a**). (**c**,**d**) Absorption spectra (red and green lines) for the diameters marked by the vertical lines in (**b**). Here, the diameter increases in the order of dashed, solid, and dashed dotted line. We show also the normalized number of available incident photons as a function of wavelength (blue line). (**e**,**f**) Photogenerated current *j*_ph1(ph2)_ in (**e**) the top cell and (**f**) the bottom cell, respectively.

It is known that the nanowire diameter affects strongly the absorption of light^1^,^5^,^7^,^8^,^13^,^35^. Therefore, to study the effect of the nanowire diameter on the absorption in the tandem cell, we fix *P* = 530 nm, *L*_top_ = 2000 nm, and *L*_bot_ = 2900 nm [Fig. 3(a)]. As a main feature: the efficiency appears to be a function of just *D*_top_ when *D*_bot_ is large enough (typically when *D*_bot_ > 250 nm). In this case of large *D*_bot_, two local maxima show up in the efficiency as a function of *D*_top_. To show these maxima clearly, we set *D*_bot_ to a fix value of 470 nm. Here, these two efficiency peaks show up at a top cell diameter of *D*_top_ = 150 nm and *D*_top_ = 345 nm, respectively [Fig. 3(b)].

To understand the origin of these two efficiency maxima, we study the number of incident photons as a function of wavelength [blue line in Fig. 3(c,d)]. In the region 600 nm to 800 nm, the solar spectrum shows the highest number of incident photons as a function of wavelength. Since we assume that each absorbed photon contributes one charge carrier to the photogenerated current, strong absorption in this wavelength region is very important for *j* and consequently to the efficiency.

Therefore, we study the absorption spectrum in the top cell as a function of the diameter in the top cell around *D*_top_ = 150 nm and *D*_top_ = 345 nm, respectively, where the two local maxima in *η* show up. In Fig. 3(c), when the diameter increases from 120 nm to 150 nm, we find an absorption peak in the spectrum, and it moves from about 600 nm to 700 nm^8^,^36^,^37^. This peak can be explained as resonant coupling of incident light into the HE_11_ waveguide mode of the individual nanowires. This resonant coupling leads to enhanced absorption in nanowire arrays^36^. When *D*_top_ increases further to 180 nm [red dotted line in Fig. 3(b)], the absorption peak has started to vanish since it red-shifts beyond the bandgap wavelength. This shifting and disappearance of the absorption peak leads consequently to a small decrease in the efficiency as *D*_top_ increases from 150 nm to 180 nm.

Similarly, in the case of *D*_top_ = 345 nm we find again an absorption peak at *λ* ≈ 700 nm [Fig. 3(d)]. This time, the absorption peak originates from the higher order HE_12_ waveguide mode. This absorption peak has red-shifted beyond the band gap wavelength when *D*_top_ has increased to 375 nm [Fig. 3(d)], leading to a slight decrease in the efficiency. Thus, we find an efficiency maximum for the nanowire tandem solar cell [Fig. 3(a)] when *D*_top_ is optimized to place the HE_11_ or the HE_12_ absorption peak just below the band gap wavelength. Very similar results have been reported for the diameter optimization of a single junction InP nanowire solar cell^8^.

To understand why the efficiency does not noticeably depend on *D*_bot_ for *D*_bot_ > 250 nm [Fig. 3(a)], we study the photogeneration of charges in the top cell (*j*_ph1_) and the bottom cell (*j*_ph2_) [Fig. 3(e,f)]. Since *j* = *j*_1_ = *j*_2_, and *j*_1_ ≤ *j*_ph1_ and *j*_2_ ≤ *j*_ph2_ (see Supplementary Information for details), the smaller one of *j*_ph1_ and *j*_ph2_ is expected to limit the solar cell efficiency [Fig. 3(a)]. When *D*_top_ < 100 nm, the total current of the tandem cell is strongly limited by *j*_ph1_. As the diameter of the top cell increases, *j*_ph1_ can increase to about 20 mA/cm^2^. However, when the bottom cell diameter is larger than 250 nm, *j*_ph2_ > 20 mA/cm^2^. Thus, for *D*_bot_ > 250 nm, *j*_ph2_ > *j*_ph1_ and the efficiency follows the absorption properties of the current-limiting top cell and therefore depends mainly on *D*_top_ and only very weakly on *D*_bot_.

We note that for the bottom cell, we find a pronounced maximum in *j*_ph2_ as a function of *D*_bot_ for *D*_bot_ ≈ 250 nm when *D*_top_ ≈ 0. We assign this maximum in *j*_ph2_ to the HE_11_ resonance in the bottom cell. We notice that in Fig. 3(f), that maximum is to a large degree overshadowed for *D*_top_ > 0 by the strong dependence of *j*_ph2_ on *D*_top_. When we study the dependence of the efficiency on *D*_bot_ for a fixed *D*_top_ (see Supplementary Figure S2), we find that the maximum at *D*_bot_ ≈ 250 nm shows up also for *D*_top_ > 0 and broadens with increasing *D*_top_.

**Figure 4:**
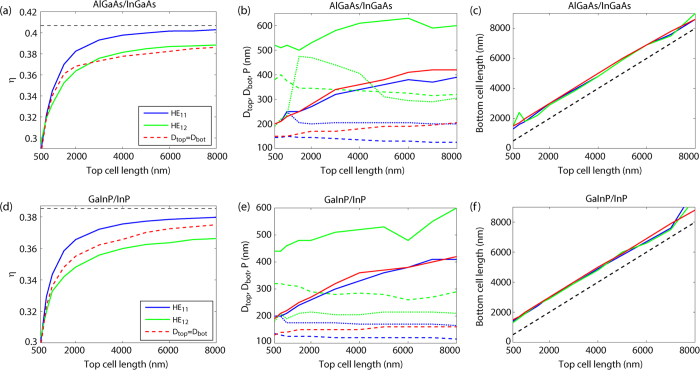
(**a**,**d**) Optimized Shockley-Queisser detailed balance efficiency as a function of *L*_top_. Here, values for the HE_11_ maximum (blue line) and the HE_12_ maximum (green line) are shown. These resonances show up when the HE_11_/HE_12_ waveguide resonance enhances absorption in the top cell for wavelengths close to the bandgap wavelength of the top cell. We show also the maximum efficiency (dashed red line) when we force a single diameter throughout the nanowire (*D*_bot_ = *D*_top_). The dashed black line shows the efficiency limit for the dual-junction cell under the assumption of perfect absorption in both the top and the bottom subcell. (**b,e**) *P* (solid line) *D*_top_ (dashed line) and *D*_bot_ (dotted line) at the maximum efficiency point. The color of the lines denotes the corresponding maximum as in (**a,d**), that is, blue for HE_11_, green for HE_12_, and red for *D*_bot_ = *D*_top_. (**c,f**) Similar as (**b,e**) but for *L*_bot_. The material of the top and the bottom subcell is shown in the title of each subfigure.

Thus, we have found above two clear local maxima for *η*, one for *D*_top_ = 150 nm and one for *D*_top_ = 345 nm that originate, respectively, from resonant absorption through the HE_11_ and HE_12_ modes in the top cell. However, the results above were derived for a fixed *L*_top_, *L*_bot_, and *P*. Next, we optimize the efficiency limit for all these five parameters (*D*_top_, *D*_bot_, *L*_top_, *L*_bot_, and *P*) simultaneously. To make the optimization numerically feasible, we introduced a numerically efficient iteration process (See Supplementary Information for details). We choose to show the results in Fig. 4 as a function of top cell length *L*_top_. For tabulated values of the optimized geometry, see Supplementary Information Table S1. For a more complete dependence of the efficiency on the geometrical parameters, see Supplementary Figures S3–S14. Notably, with proper design, an efficiency limit above 40% can be reached by the use of Al_0.10_Ga_0.90_As for the top cell and In_0.34_Ga_0.66_As for the bottom cell [blue line, when *L*_top_ > 6 μm, in Fig. 4(a)].

In this optimization, we can identify maxima in *η* to originate from the above discussed HE_11_ and HE_12_ resonances in the top cell [Fig. 4(a)]. In the region of *L*_top_ > 600 nm, the HE_11_ resonance of the top cell leads to a higher efficiency limit than that of the HE_12_ resonance. These results are in agreement with those for a single junction nanowire array solar cell where the HE_11_ resonance usually leads to the highest efficiency^8^. For the dual junction cell here, we call these maxima for brevity the HE_11_ and HE_12_ maxima/optima.

For a single junction nanowire cell^8^, rough values for the optimum diameter were estimated as


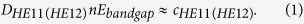


Here, 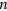 is the real part of the refractive index (at the band gap wavelength) and *D*_HE11(HE12)_ is the diameter that optimizes the wavelength position of the HE_11_ and HE_12_ resonance in order to maximize *η*. The value for the constant *c*_HE11(12)_ can be extracted from the work on the single-junction nanowire solar cells^8^.

The diameter for the HE_11_ (HE_12_) resonance of the top cell in Fig. 4(b) is *D*_top_ ≈ 150 nm (*D*_top_ ≈ 345 nm) in qualitative agreement with values from Equation (1) [about 169 nm for HE_11_ and 394 nm for HE_12_ resonance]. We find that *D*_bot_ fluctuates only slightly when *D*_top_ ≈ 150 nm to yield the HE_11_ maximum (blue dotted line in Fig. 4(b)). In contrast, *D*_bot_ fluctuates more at the HE_12_ maximum (green dotted line in Fig. 4(b)). This fluctuation in *D*_bot_ is understood from the fact that for the HE_12_ maximum at *D*_top_ ≈ 345 nm, the efficiency shows a very broad maximum in *D*_bot_ (black dashed line in Fig. 3a and Supplementary Figure S2), which allows for large variations in *D*_bot_ when *L*_top_, *L*_bot_, and *P* are optimized.

Similarly as for the single nanowire case^8^, we find that the optimum pitch *P* [solid lines in Fig. 4(b)] tends to increase with increasing nanowire length, that is, with increasing *L*_top_ and *L*_bot_. This behavior can be understood as a competition between increased absorption and increased reflection with decreasing *P*^8^. With increasing nanowire length, the absorption increases, and we can allow for a larger *P* to decrease reflection losses.

In our results, we find that *L*_bot_ > *L*_top_ [Fig. 4(f)]. However, the efficiency tends to increase as a function of *L*_bot_ (see Supplementary Figures S3–S14), and therefore whether we end up in the case of *L*_bot_ > *L*_top_ or in the case of *L*_bot_ < *L*_top_ depends on how heavily we maximize the efficiency *η* at the cost of increasing *L*_bot_. We allowed the optimization to stop with respect to *L*_bot_ when we reached a value of d*η*/d*L*_bot_ < 0.001 μm^−1^ in our geometry optimization (see Supplementary Information). In this case, for all the considered *L*_top_, the optimized value for *L*_bot_ ended up slightly larger than *L*_top_.

For fabrication purposes, it could be a benefit to consider *D*_top_ = *D*_bot_, that is, nanowires of a single diameter *D* throughout (see the red dashed line in Fig. 4a for the resulting efficiency). We found in this case large fluctuations in the optimum value of *L*_bot_ when *L*_top_ is increasing (the fluctuation in *L*_bot_ could be larger than the value of *L*_top_). To be able to analyze this case as a function of *L*_top_, we set an upper limit of *L*_top_+1000 nm for *L*_bot_.

We find an interesting behavior for the optimized diameter *D* for these single-diameter nanowires [red dashed line in Fig. 4(b)]. For the smallest considered *L*_top_ of 500 nm, *D* starts close to the *D*_top_ ≈ *D*_HE11_ ≈ 150 nm of the HE_11_ maximum for the case in which we allow for *D*_top_ ≠ *D*_bot_. When *L*_top_ increases toward the largest considered value of 8000 nm, *D* increases toward the value of the *D*_bot_ ≈ 200 nm which optimizes the HE_11_ maximum in the *D*_top_ ≠ *D*_bot_ case. This behavior can be understood as follows. When *L*_top_ is small, the absorption in the top cell is weak in relative terms, and photons also in the short wavelength region can reach the bottom cell due to insufficient absorption in the top cell. As a result, the current, and therefore the efficiency, of the solar cell is limited by absorption in the top cell. As a consequence, the optimum *D* occurs when the absorption in the top cell is optimized for, which happens at *D* ≈ *D*_HE11_. In contrast, when *L*_top_ is large, the absorption in the top cell is instead strong, and the performance of the solar cell becomes limited by the current-generation in the bottom cell, which is optimized for *D* in a similar way as when *D*_top_ ≠ *D*_bot_. Thus, for large *L*_top_, *D* goes toward the *D*_bot_ that optimizes the HE_11_ maximum.

Since we find the optimum for *D* close to the diameters found for the HE_11_ maximum in the *D*_top_ ≠ *D*_bot_ case, we find, not completely surprisingly, values for *P* close to those of the HE_11_ case of *D*_top_ ≠ *D*_bot_. As an end result, we find that the efficiency for this case of *D* = *D*_top_ = *D*_bot_ is typically 1 to 2% lower than when we allow for *D*_top_ ≠ *D*_bot_ [Fig. 4(a)].

We have also studied the efficiency of the InP based Ga_0.51_In_0.49_P/InP nanowire tandem system [Fig. 4(d–f)], with maximum efficiency of 38.5% for perfectly absorbing subcells, which should be set in relation to the limit of 42.0% for the idealized, perfectly absorbing, band gap optimized dual-junction bulk cell. Also for this material choice we reach an efficiency within 2% of this maximum, with *L*_top_ > 6 μm and *L*_bot_ > 7 μm, when we allow for *D*_top_ ≠ *D*_bot_. Also here, an additional drop by about 1% occurs with the constraint *D*_top_ = *D*_bot_. With Ga_0.51_In_0.49_P and InP as the material and *D*_top_ = *D*_bot_, we reach *η* = 35.5% when *L*_top_ = 2000 nm and *L*_bot_ = 3000 nm, considerably higher than the maximum 31.0% possible in the single junction bulk solar cell case. To aid the reader, we show in Table 1 the values extracted from Fig. 4 for this case of *D*_top_ = *D*_bot_ (for the HE_11_ and HE_12_ maximum, we refer the reader to Supplementary Information Table S1).

## Conclusion

We performed electromagnetic modeling to investigate theoretically the absorption properties of a dual junction nanowire array solar cell. We used then the Shockley-Queisser efficiency limit as a metric for optimizing the materials and geometry of the nanowires. The optimized geometries are presented in Fig. 4, Table 1, and Supplementary Information Table S1. The drop in efficiency limit when moving away from such an optimized geometry is presented in Supplementary Information Figures S3–S14. These results present a guideline for choosing a nanowire geometry that has promise for optimized absorption in a dual-junction nanowire array solar cell. In this way, our results can be used as a starting point for theoretical studies on the optimization of the electrical properties of dual-junction nanowire array solar cells. Our results can also guide in the choice of materials and dimensions for the fabrication of nanowires aimed for tandem solar cells.

## Additional Information

**How to cite this article**: Chen, Y. *et al.* Design for strong absorption in a nanowire array tandem solar cell. *Sci. Rep.* **6**, 32349; doi: 10.1038/srep32349 (2016).

## Supplementary Material

Supplementary Information

## Figures and Tables

**Table 1:** Optimized efficiency for varying *L*_top_ for the Ga_0.51_In_0.49_P/InP dual-junction solar cell when the nanowire has a single diameter (*D*_top_ = *D*_bot_), together with the corresponding geometrical parameters, as extracted from Fig. 4(d–f).

*L*_top_ (nm)	*D*_top_ (nm)	*D*_bot_ (nm)	*L*_bot_ (nm)	*P* (nm)	*η*(%)
500	130	130	1500	190	29.7
1000	140	140	2000	220	33.6
2000	150	150	3000	270	35.5
4000	150	150	5000	360	36.6
8000	160	160	8800	420	37.5
