# Supplementary material for: Design for strong absorption in a nanowire array tandem solar cell
Source: Sci Rep. 2016 Aug 30;6:32349. doi: 10.1038/srep32349 (PMC5004118; doi:10.1038/srep32349)
Supplement: Supplementary Information [file srep32349-s1.pdf]

Supplementary Information:

Design for strong absorption in a nanowire array tandem solar cell

Yang Chen, Mats-Erik Pistol, and Nicklas Anttu*

Division of Solid State Physics and NanoLund,
Lund University,
Box 118, 22100 Lund, Sweden
**nicklas.anttu@ftf.lth.se*

Direct iteration method to optimize geometry

In general, for an optimization problem of a function η with variables x_1, x_2 ...x_n, (such as the L_{bot}, D_{top}, D_{bot}, P, and L_{top} in the case of our nanowire array), the necessary condition for a maximum point is $\forall\ i,\ \frac{\partial \eta}{\partial x_i} = 0,$ unless the maximum resides at the boundary of the independent variable domain.

Many of the conventional optimization methods, such as the Newton's method, are based on first and/or second order derivatives. However, the efficiency η of the dual-junction nanowire solar cell shows a dependence on L_{top}, L_{bot}, D_{top}, D_{bot}, and P that makes the optimization to appear unsuitable for such derivative based methods. First, the efficiency tends to increase monotonously with both L_{top} and L_{bot} (see Figures S3-S14). Second, for a fixed L_{top} and L_{bot}, the efficiency can show a rugged landscape with many local maxima as a function of D_{top}, D_{bot}, and P (see Figures S3-S14).

Here, we use a direct iteration method where we vary one geometrical parameter at a time. We choose to perform the iteration for a fixed L_{top}. Regarding the L_{bot}, for which η tends to increase monotonously, we allow the iteration to stop if $d\eta/dL_{bot} < 0.001$ μm^{-1} in the iteration. See Algorithm 1 below for a brief pseudocode description of the iteration method.

Algorithm 1

```
Choose L_top to solve for.
Define variable names as follows: x1 = D_top, x2 = D_bot, x3 = P, x4 = L_bot
Define current point (x1,c, x2,c, x3,c, x4,c) %starting point for iteration

η_prev =0    %efficiency value in previous iteration step
optimum_not_found=1
While(optimum_not_found)
   x4,prev=x4,c  %to find derivative dη/dx4 = dη/dL_bot for which we set a limit of 0.001 μm^-1

   For i=1 to 4
      Define parameter limits, xi,lb ≤ xi ≤ xi,ub, and step length xi,step
      %typically D_top < P, D_top,step = 5 nm
      %typically D_bot < P, D_bot,step = 5 nm
      %typically P < 2000 nm, P_step = 10 nm
      %typically L_bot < 10000 nm, L_bot,step = 100 nm
      η_max =0   %maximum value of efficiency found in iteration over xi
      For xit=xi,lb to xi,ub in step of xi,step
         Compute A1 (λ) and A2 (λ) for λ < λ2 with xi = xit (using xj = xj,c for j ≠ i)
         Compute efficiency η for A1 (λ) and A2 (λ) %Equations (S1)-(S6)
         If η> η_max
            η_max=η
            xi,c=xit   %updates current optimized geometry point --- affects iteration when moving from i to i+1 in for loop)
         end
      end
      ηi = η_max  %stores efficiency from iteration over xi
   end
   if (η1 – η_prev)<0.0001 && (η2 – η_prev)<0.0001 && (η3 – η_prev)<0.0001 && (η4 – η_prev)/(x4,c – x4,prev)< 0.001*1e6
      optimum_not_found=0  %ends iteration since increase in eta smaller than set limit
   end
   η_prev = η4
end
%η_prev contains now the maximum efficiency after iteration and (x1,c, x2,c, x3,c, x4,c) optimized geometry
```

Efficiency analysis

The most important property of a solar cell is η, the conversion efficiency of sun light into electrical energy. To theoretically study η, the Shockley-Queisser detailed balance analysis gives a general framework. There, a balance between photo generation of electron-hole pairs, extracted current, and recombination of electron-hole pairs is used[1-3]. This analysis is of general character and is applicable also for p-i-n junction tandem solar cells[3-5]. By considering optical absorption and radiative recombination, we find an upper limit for the efficiency of a solar cell design[2,5-9]. Non-radiative recombination and ohmic losses reduce the efficiency from this radiatively limited efficiency[3,6,10]. In this way, we study the prospect of the nanowire tandem solar cell as a platform for next-generation photovoltaics, in the case when material properties, such as non-radiative recombination and ohmic losses, are minimized.

Let us consider a dual-junction tandem solar cell where we by cell 1 denote the top cell and by cell 2 the bottom cell (Figure 1). For such a solar cell under AM1.5D solar illumination, the current density as a function of voltage in each subcell is given by[1-3]

$$j_{1(2)} = j_{ph1(ph2)} - j_{rec1(rec2)}(V_{1(2)}). \tag{S1}$$

Here, $j_{1(2)}$ is the total current in the top (bottom) cell, $j_{\mathrm{ph1(ph2)}}$ is the photogenerated current density under the AM1.5D solar spectrum, and $j_{rec1(\mathrm{rec2})}$, which depends on the voltage $V_{1(2)}$ over subcell 1(2), is the decrease of current due to the varying types of recombination losses. We consider here a series connected tandem cell where the same current $j = j_1 = j_2$ flows through both cells. Note that in this case of a series-connected subcells, the voltages V_1 and V_2 must be coupled in such a way that the condition $j_1 = j_2$ is fulfilled. The output power of the tandem cell is given by $P = (V_1+V_2)j$. Note that this power is maximized at $V_1 = V_{1\mathrm{max}}$ and $V_2 = V_{2\mathrm{max}}$, where the current is $j = j_{\mathrm{max}}$ and the output power $P = P_{\mathrm{max}} = (V_{1\mathrm{max}} + V_{2\mathrm{max}})\, j_{\mathrm{max}}$. Note that in this analysis, we vary both V_1 and V_2 in order to find $V_{1\mathrm{max}}$ and $V_{2\mathrm{max}}$, under the constraint that $j_1 = j_2$.

The Shockley-Queisser detailed balance efficiency, η, is in turn given by the ratio between this P_{max} and the total incident solar power[1]:

$$\eta = \frac{(V_{1max}+V_{2max})j_{max}}{\int_0^\infty I_{AM1.5}(\lambda)d\lambda}. \tag{S2}$$

Here, $I_{\mathrm{AM1.5}}(\lambda)$ is the 1 sun direct and circumsolar AM1.5D solar spectrum[5].

We start by considering the photogenerated current $j_{\mathrm{ph1(ph2)}}$ in each cell. Here, we assume that each absorbed photon with energy above the band gap energy generates one electron-hole pair in the solar cell. Notice that each

photogenerated electron-hole pair contributes one charge carrier to this current since the electron and hole are split by the p-i-n junction in different directions[3,9]. The incident intensity multiplied by the absorption probability $A(\lambda)$ gives the amount of absorbed energy at wavelength λ. By dividing this absorbed energy with $2\pi\hbar c/\lambda$, we obtain the rate of photons absorbed at energy λ. By integrating this rate over wavelength, we find the generation rate of electron-hole pairs, and by multiplying with the elementary charge e, we find the photogenerated current (density):

$$j_{ph1(ph2)} = \frac{e}{2\pi\hbar c}\int_0^{\lambda_{1(2)}} d\lambda\left[\lambda I_{AM1.5}(\lambda)A_{1(2)}(\lambda)\right] \qquad \text{(S3)}$$

Here, $\lambda_{1(2)}=\mathrm{hc}/E_{1(2)}$ corresponds to the wavelength of photons at the band gap energy $E_{1(2)}$ of cell 1 and 2, respectively, and c is the speed of light in vacuum. We assume normally incident light, to maximize the projected area of the solar cell to the incident light. Thus, $A_{1(2)}(\lambda)$ is the absorptance of normally incident light in the top (bottom) cell. Note that an absorbed photon is absorbed either in the top or the bottom cell, which leads to the condition $0 \leq A_1(\lambda) + A_2(\lambda) \leq 1$. We allow for the realistic case of incomplete absorption of photons in either subcell. For example, high energy photons can reach the bottom cell due to $A_1(\lambda) < 1$ and contribute to photogeneration in the bottom cell. Note that we in Eq. (S3) neglect the re-absorption of photons emitted from the other subcell. Such re-absorption between subcells is expected to contribute only 0.1-0.2% to the Shockley-Queisser detailed balance efficiency for optimized cells[1].

Now that we know how to analyze $j_{ph1(ph2)}$, we turn to consider the radiative recombination. Here, since we consider the case of a solar cell limited by the radiative recombination, $j_{rec1(rec2)} = j_{rad1(rad2)}$ in Eq. (S1). In a semiconductor solar cell, the radiative recombination is typically given by[2,3,6]

$$j_{rad1(rad2)}(V_{1(2)}) = e(F_{c0,up,1(2)} + F_{c0,down,1(2)})[\exp\left(\frac{eV_{1(2)}}{k_bT}\right) - 1] \qquad \text{(S4)}$$

where the radiative emission rate at thermal equilibrium is given by:

$$F_{c0,up(down),1(2)} = \int_0^{2\pi} d\varphi \int_0^{\pi/2} d\theta \int_0^{\lambda_{1(2)}} d\lambda \left[\frac{cn^2_{up(down),1(2)}}{\lambda^4\left(\exp\left(\frac{2\pi\hbar c}{\lambda k_bT}\right)-1\right)}(e_{TE,up(down),1(2)}(\lambda,\theta,\varphi) + e_{TM,up(down),1(2)}(\lambda,\theta,\varphi))cos\theta sin\theta\right]. \qquad \text{(S5)}$$

The subscripts 1 and 2 denote, as before, the top and the bottom subcell. In contrast, the subscripts *up* and *down* denote the upper and lower surface/side of each subcell. For example, $n_{up,1}$ is the real part of the refractive index of the material on the upper side of the top cell, and $e_{TE,down,2}(\lambda,\theta,\varphi)$ is the emissivity of TE polarized light to the bottom side from the bottom cell. Thus, the emission from the nanowire solar cell depends on the emissivity of

each subcell to the top and the bottom side. In principle, the nanowire geometry can affect the emission properties of the solar cell, and therefore also the efficiency limit. For example, for a single-junction solar cell, an absolute increase in the efficiency limit by 1-2% has been predicted compared with planar cells[2]. However, an analysis of the emission properties of nanowire tandem solar cells is beyond the scope and computational resources of this work. Instead, we believe that by using the simplifying approximation $e_{\mathrm{TE/TM,up(down),1(2)}}$ =1, we obtain a reasonable comparison between solar cells constructed from different nanowire geometries and materials. In this way, we assume maximum possible emission from the solar cell. Notice that this assumption gives a lower bound for the efficiency of the solar cell in the radiative limit. With this assumption of e =1, the radiative recombination rate at thermal equilibrium simplifies to:

$$F_{c0,up(down),1(2)} = 2\pi c \int_0^{\lambda_{1(2)}} d\lambda \left[\frac{\tilde{n}^2_{up(down),1(2)}}{\lambda^4(\exp(2\pi\hbar c/\lambda k_b T)-1)}\right]. \qquad \text{(S6)}$$

Here, $\tilde{n}_{\mathrm{up(down),1(2)}}$ is the effective refractive index for emission and depends on how light can couple out from the top and the bottom cell to the top air side and into the substrate[11]. We assume that the emission into the substrate can occur from the top and bottom cell at all angles (see Figure S1(a)). This gives $\tilde{n}_{\mathrm{down,1(2)}} = n_{\mathrm{substrate}}$. For the emission into the top air side, we are limited to propagation angles $\theta < 90^0$ in the air. This limitation on emission angles translates into $\tilde{n}_{\mathrm{up,1(2)}} = n_{\mathrm{air}} = 1$ in Eq. (S6)[11].

Table S1. Optimized geometry dimensions, as extracted from Figure 4, and resulting maximized efficiency limit.

		L_{top} (nm)	D_{top} (nm)	D_{bot} (nm)	L_{bot} (nm)	P (nm)	η (%)
$Al_{0.10}Ga_{0.90}As$ $In_{0.34}Ga_{0.66}As$	HE_{11}	500	145	200	1300	200	28.6
		1000	150	250	1800	250	34.5
		2000	145	200	3000	270	38.3
		4000	135	205	4800	340	39.8
		8000	125	200	8600	390	40.3
	HE_{12}	500	380	190	1500	520	29.4
		1000	375	235	1800	520	33.2
		2000	345	470	2900	530	36.4
		4000	335	405	4800	610	38.1
		8000	320	305	9000	600	38.8
	Single *D*: $D_{top} = D_{bot}$	500	150	150	1500	200	28.2
		1000	150	150	2000	230	34.0
		2000	170	170	3000	280	36.8
		4000	180	180	5000	360	37.8
		8000	205	205	8600	420	38.6
$Ga_{0.51}In_{0.49}P$ InP	HE_{11}	500	135	200	1400	200	30.0
		1000	125	175	1900	210	34.4
		2000	125	175	3000	260	36.6
		4000	120	170	4900	330	37.6
		8000	115	165	10200	410	38.0
	HE_{12}	500	320	180	1300	440	30.4
		1000	315	190	1900	460	33.3
		2000	290	215	2900	480	34.8
		4000	285	205	4800	520	36.0
		8000	290	210	9600	600	36.6
	Single *D*: $D_{top} = D_{bot}$	500	130	130	1500	190	29.7
		1000	140	140	2000	220	33.6
		2000	150	150	3000	270	35.5
		4000	150	150	5000	360	36.6
		8000	160	160	8800	420	37.5

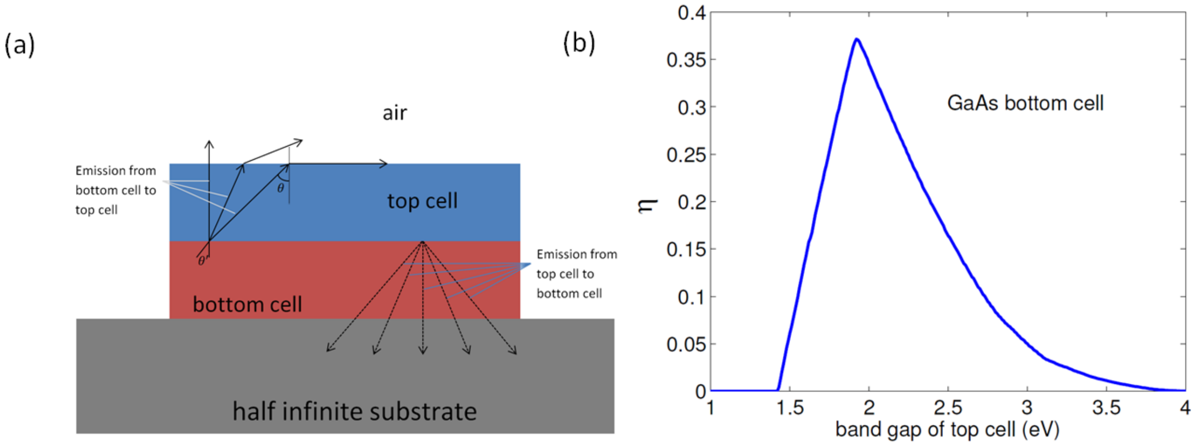

Figure S1. (a) Schematic diagram of light refraction in a dual-junction solar cell. (b) Shockley-Queisser detailed balance efficiency as a function of material band gaps for perfectly absorbing subcells. Here, GaAs is fixed as the bottom cell material. In this case, the best bandgap for the top cell is 1.92 eV with an efficiency limit of η = 37.2%.

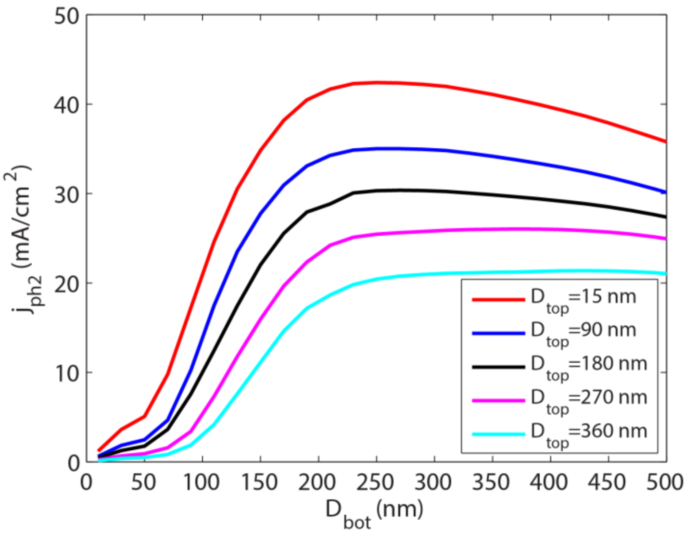

Figure S2. Line cuts from Figure 3(f) in the main text for fixed D_{top}. That is, the photogenerated current in cell 2, the bottom cell, as a function of D_{bot} for P = *530* nm, L_{top} = 2000 nm, and L_{bot} = 2900 nm. Here, the results for D_{top} = 15, 90, 180, 270, and 360 nm are shown.

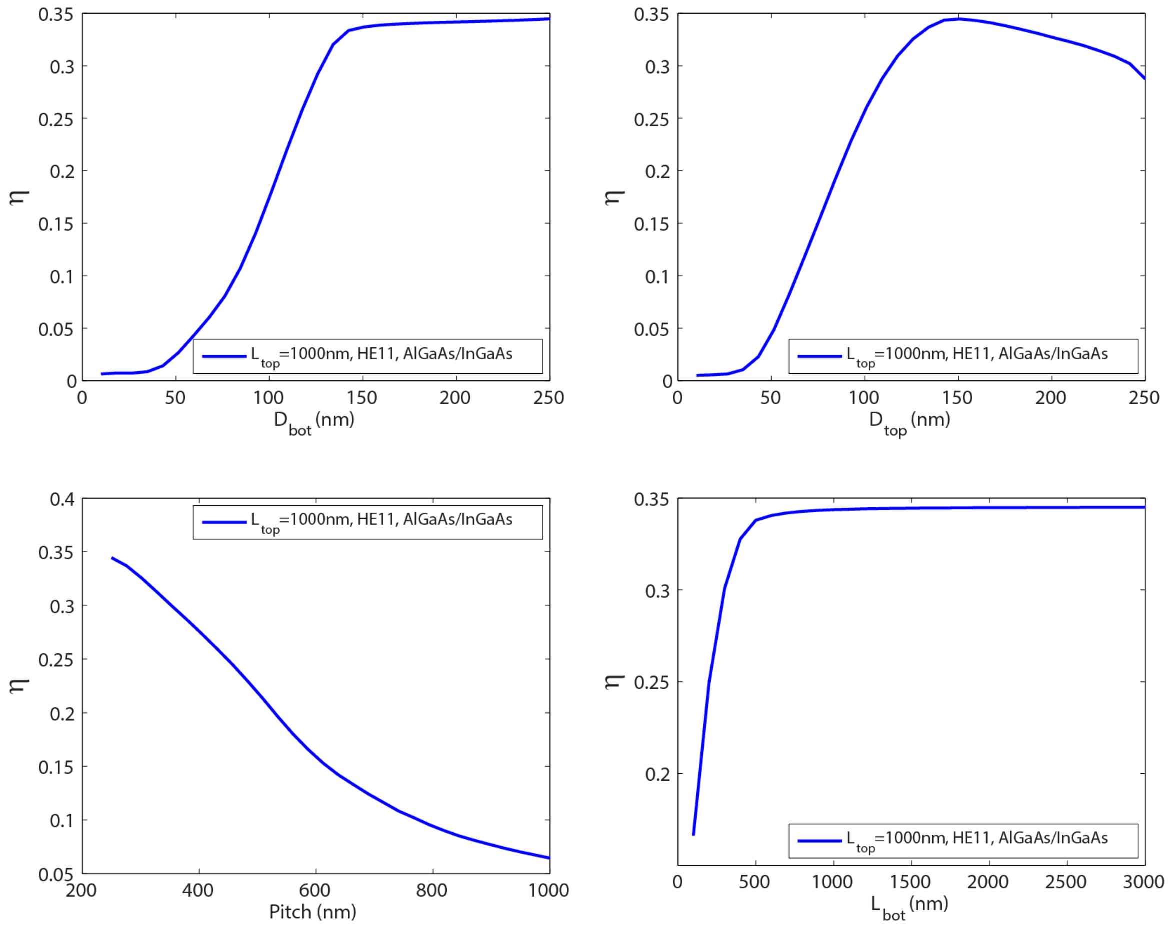

Figure S3. Dependence of the efficiency on D_{bot}, D_{top}, P, and L_{bot} when L_{top} = 1000 nm for the HE_{11} maximum in the AlGaAs/InGaAs dual junction nanowire solar cell. For the separate variation of each parameter shown here, the other three parameters are kept at their optimized value (D_{bot} = 150 nm, D_{top} = 250 nm, P = 250 nm, and L_{bot} = 1800 nm give the optimized η = 34.5 %; see Table S1).

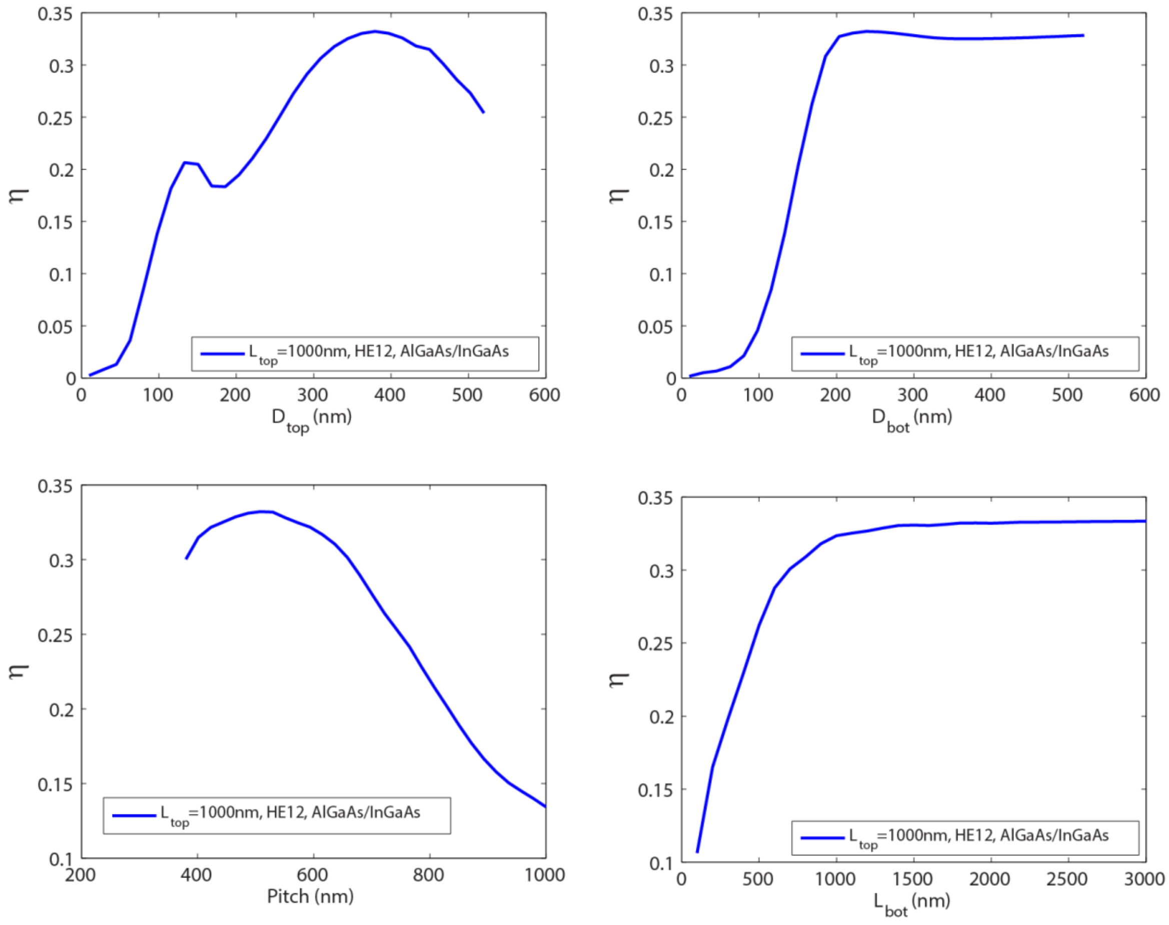

Figure S4. Dependence of the efficiency on D_{bot}, D_{top}, P, and L_{bot} when L_{top} = 1000 nm for the HE_{12} maximum in the AlGaAs/InGaAs dual junction nanowire solar cell. For the separate variation of each parameter shown here, the other three parameters are kept at their optimized value (D_{bot} = 375 nm, D_{top} = 235 nm, P = 520 nm, and L_{bot} = 1800 nm give the optimized η = 33.2 %; see Table S1).

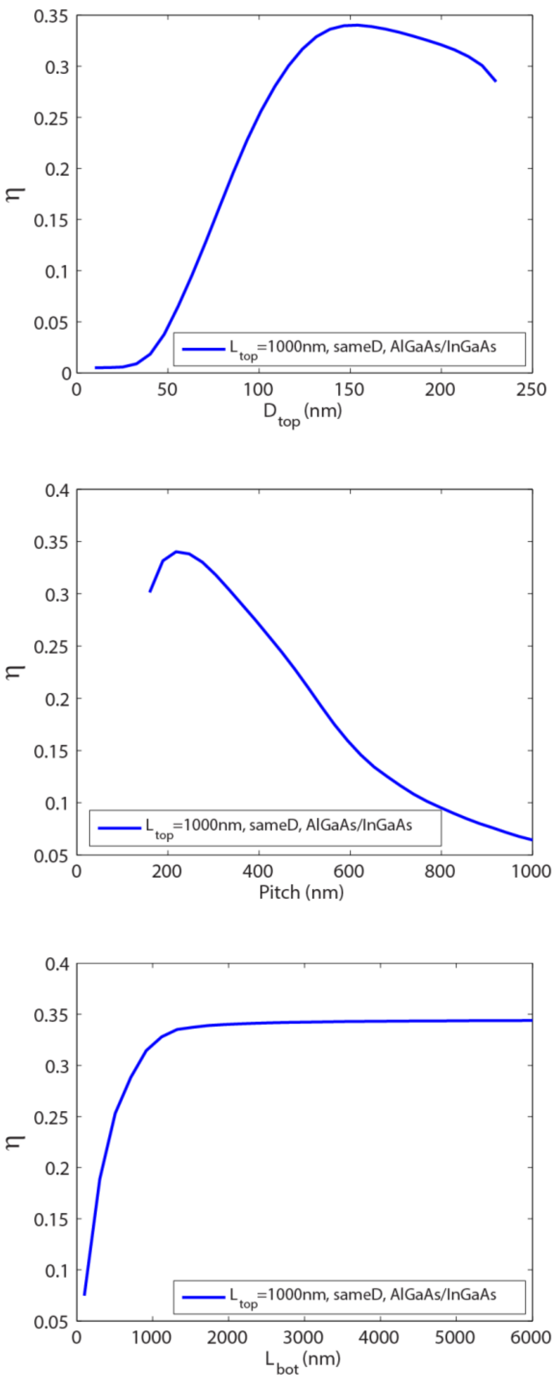

Figure S5. Dependence of the efficiency on $D_{bot} = D_{top}$, P, and L_{bot} when L_{top} = 1000 nm for the AlGaAs/InGaAs dual junction nanowire solar cell. For the separate variation of each parameter shown here, the other three parameters are kept at their optimized value ($D_{bot} = D_{top}$ = 150 nm, P = 230 nm, and L_{bot} = 2000 nm give the optimized η = 34.0 %; see Table S1).

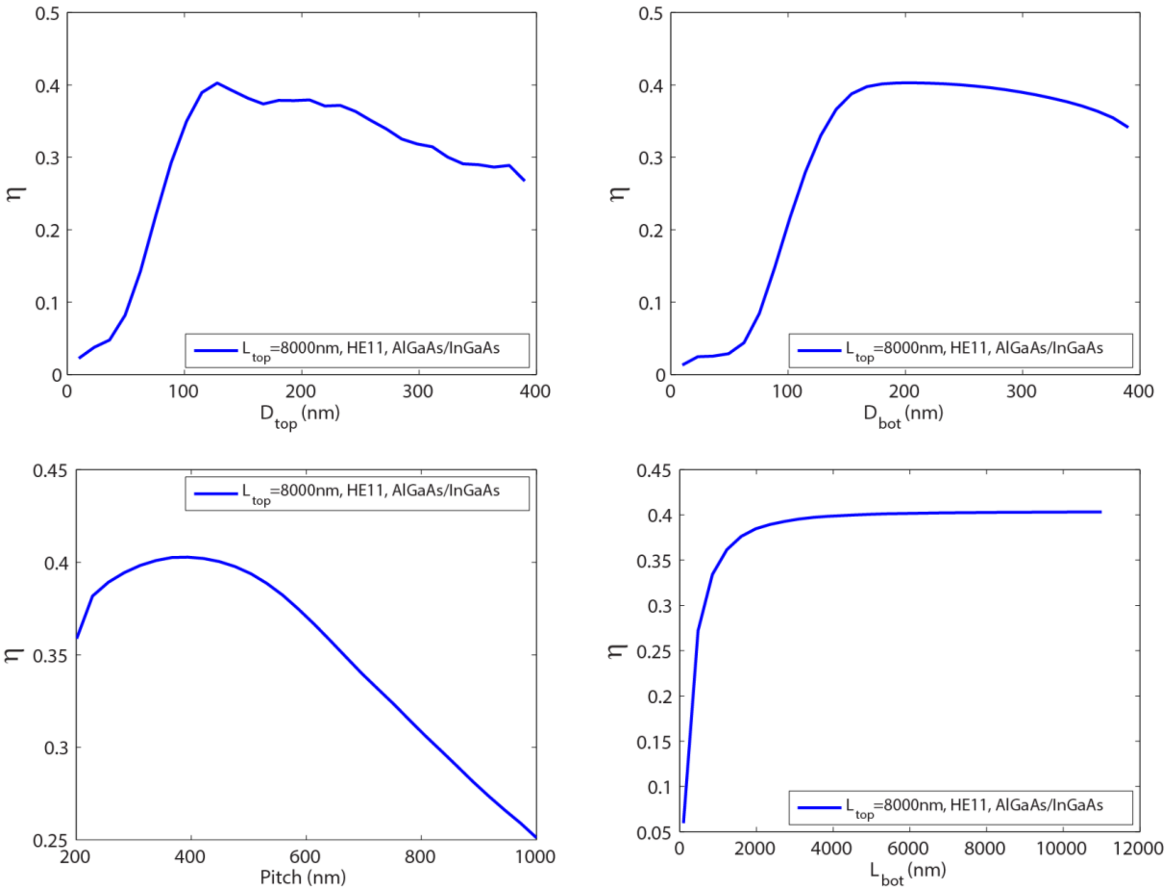

Figure S6. Dependence of the efficiency on D_{bot}, D_{top}, P, and L_{bot} when L_{top} = 8000 nm for the HE_{11} maximum in the AlGaAs/InGaAs dual junction nanowire solar cell. For the separate variation of each parameter shown here, the other three parameters are kept at their optimized value (D_{bot} = 125 nm, D_{top} = 200 nm, P = 390 nm, and L_{bot} = 8600 nm give the optimized η = 40.3 %; see Table S1).

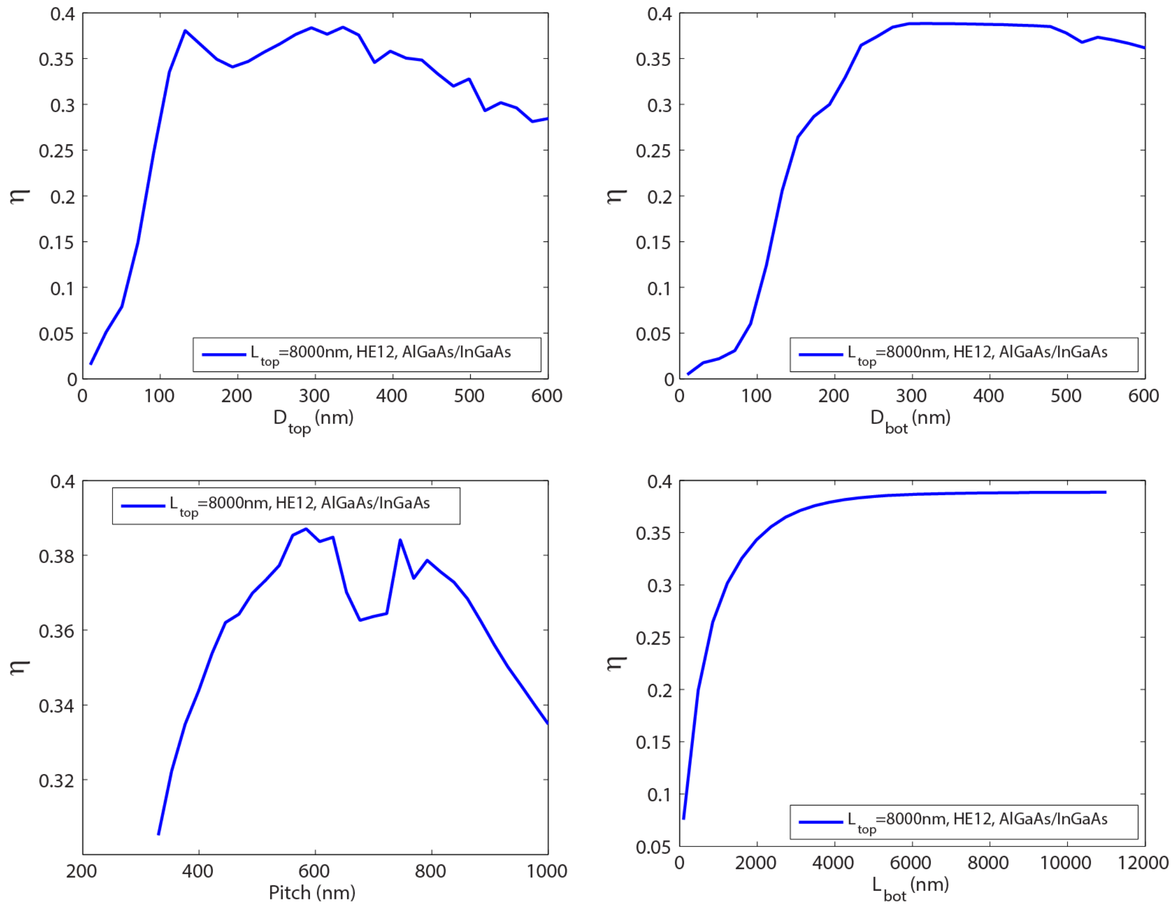

Figure S7. Dependence of the efficiency on D_{bot}, D_{top}, P, and L_{bot} when L_{top} = 8000 nm for the HE_{12} maximum in the AlGaAs/InGaAs dual junction nanowire solar cell. For the separate variation of each parameter shown here, the other three parameters are kept at their optimized value (D_{bot} = 320 nm, D_{top} = 305 nm, P = 600 nm, and L_{bot} = 9000 nm give the optimized η = 38.8 %; see Table S1).

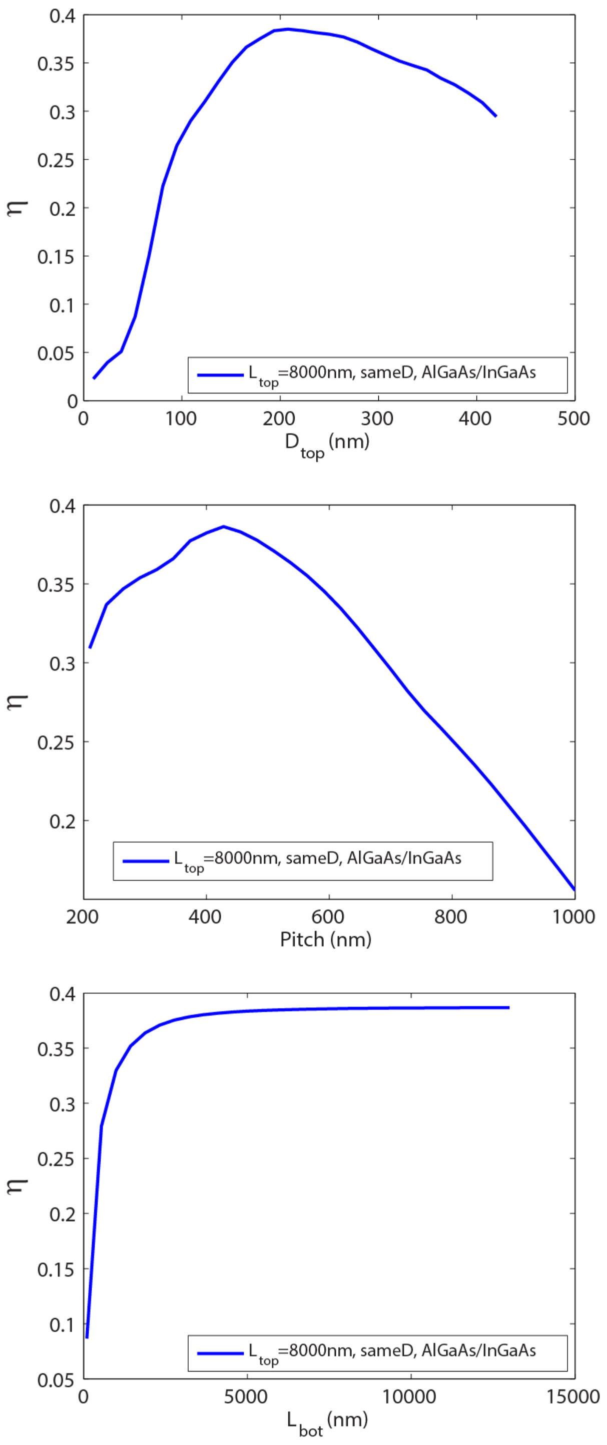

Figure S8. Dependence of the efficiency on $D_{bot} = D_{top}$, P, and L_{bot} when L_{top} = 8000 nm for the AlGaAs/InGaAs dual junction nanowire solar cell. For the separate variation of each parameter shown here, the other three parameters are kept at their optimized value (D_{bot} = D_{top} = 205 nm, P = 420 nm, and L_{bot} = 8600 nm give the optimized η = 38.6 %; see Table S1).

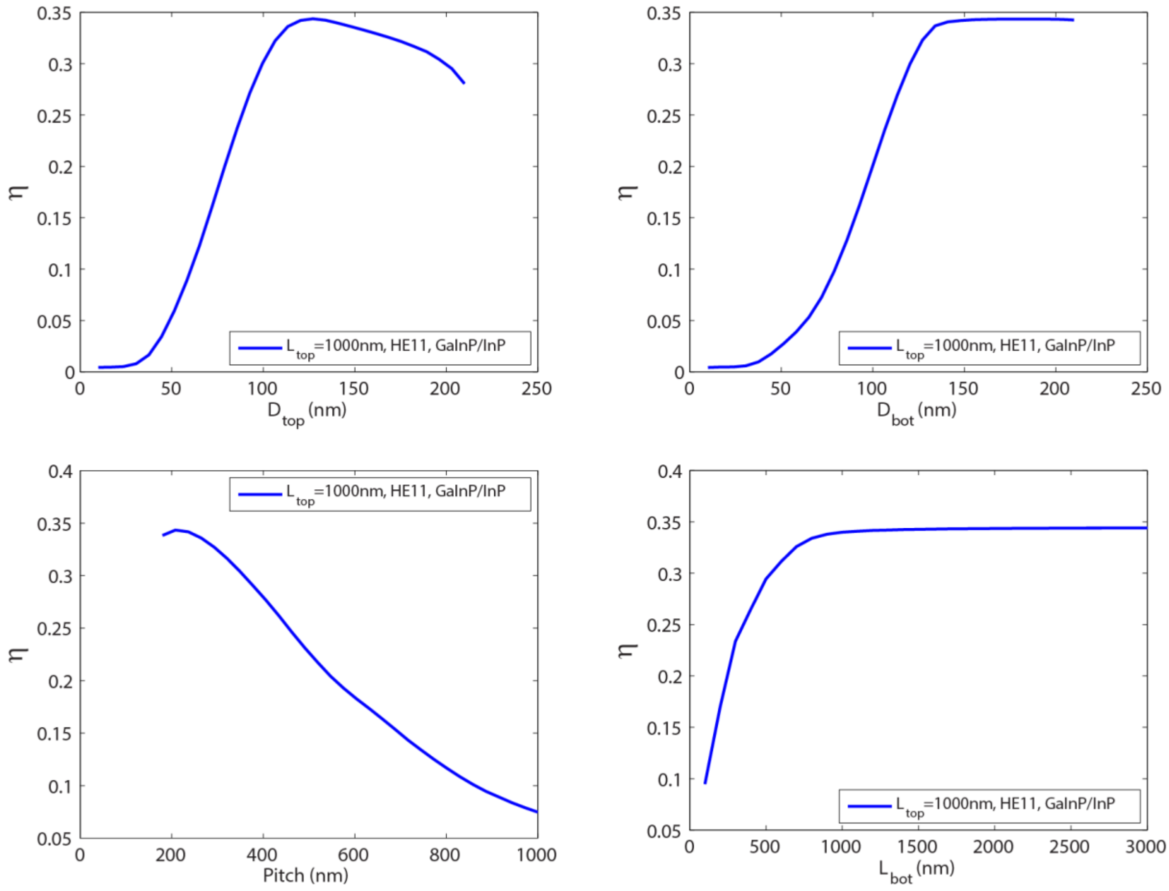

Figure S9. Dependence of the efficiency on D_{bot}, D_{top}, P, and L_{bot} when L_{top} = 1000 nm for the HE_{11} maximum in the GaInP/InP dual junction nanowire solar cell. For the separate variation of each parameter shown here, the other three parameters are kept at their optimized value (D_{bot} = 125 nm, D_{top} = 175 nm, P = 210 nm, and L_{bot} = 1900 nm give the optimized η = 34.4 %; see Table S1).

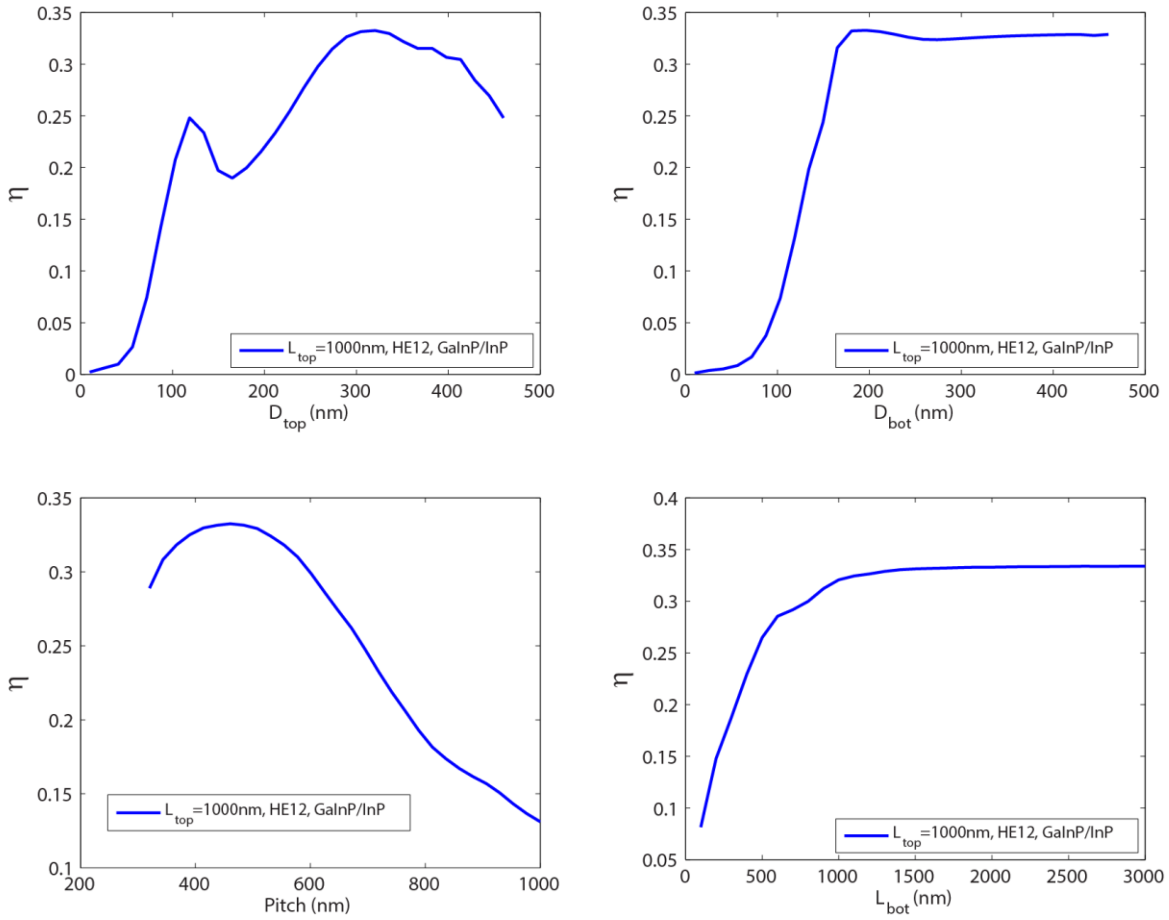

Figure S10. Dependence of the efficiency on D_{bot}, D_{top}, P, and L_{bot} when L_{top} = 1000 nm for the HE_{12} maximum in the GaInP/InP dual junction nanowire solar cell. For the separate variation of each parameter shown here, the other three parameters are kept at their optimized value (D_{bot} = 315 nm, D_{top} = 190 nm, P = 460 nm, and L_{bot} = 1900 nm give the optimized η = 33.3 %; see Table S1).

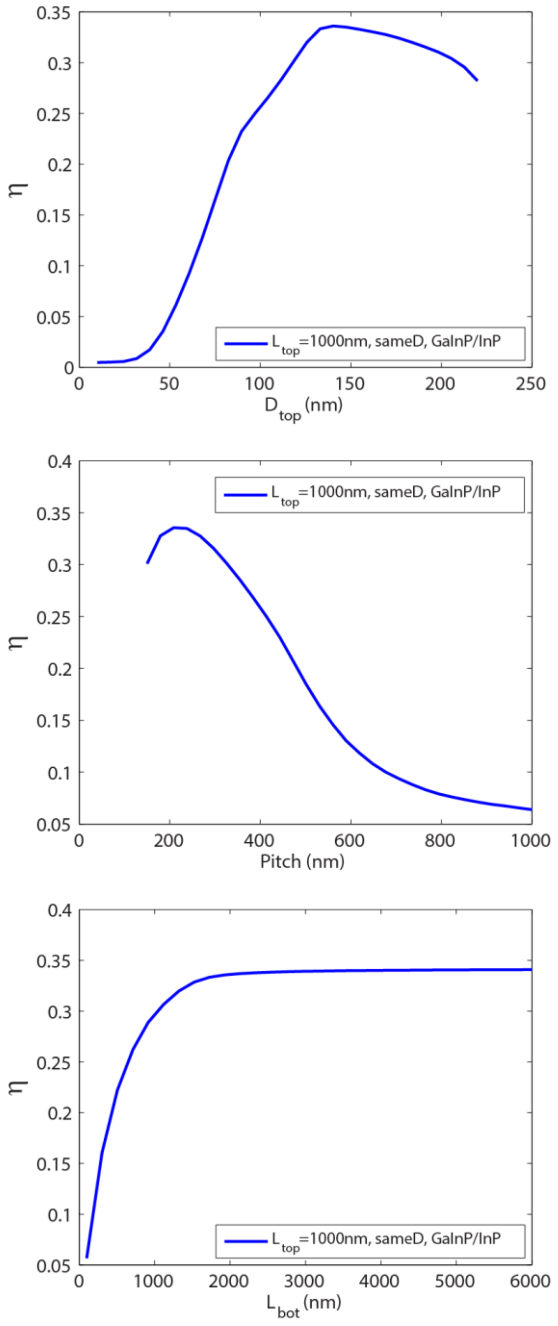

Figure S11. Dependence of the efficiency on $D_{bot} = D_{top}$, P, and L_{bot} when L_{top} = 1000 nm for the GaInP/InP dual junction nanowire solar cell. For the separate variation of each parameter shown here, the other three parameters are kept at their optimized value ($D_{bot} = D_{top}$ = 140 nm, P = 220 nm, and L_{bot} = 2000 nm give the optimized η = 33.6 %; see Table S1).

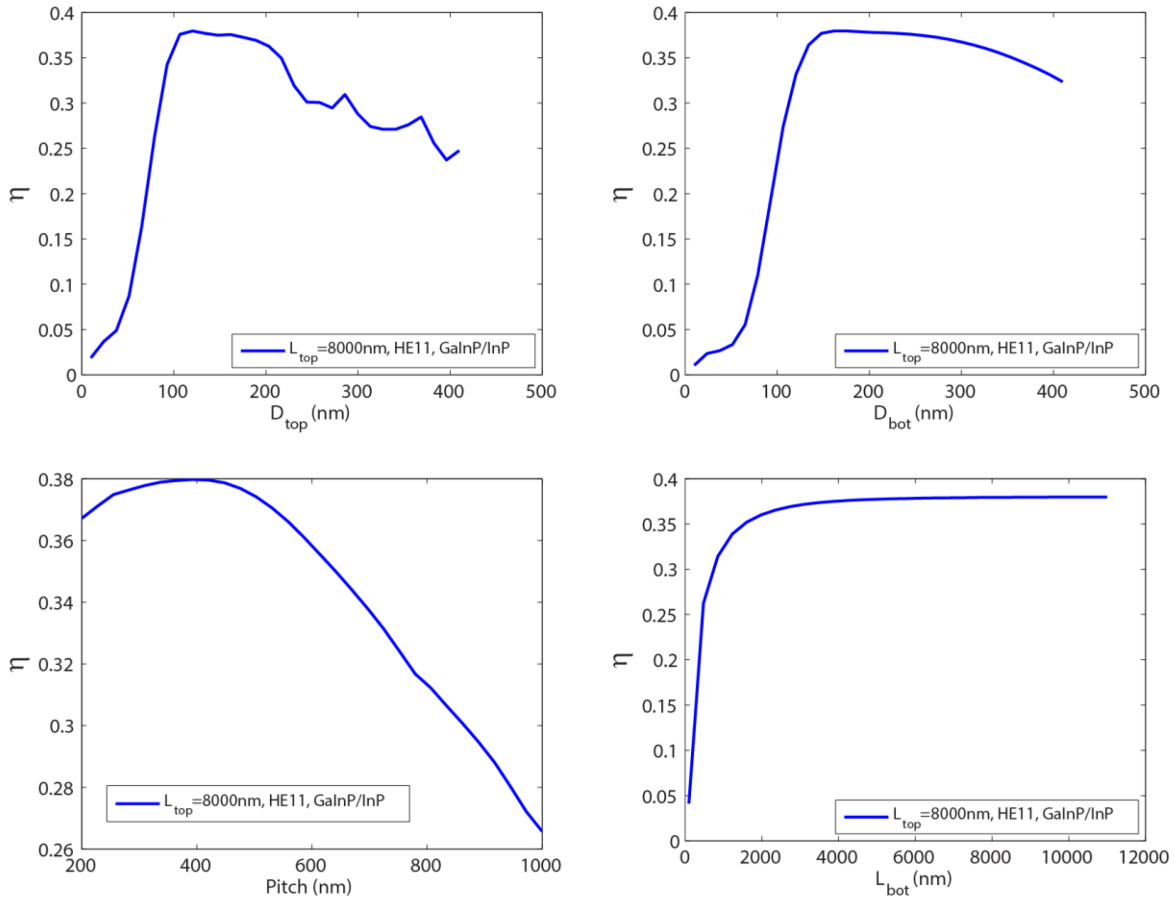

Figure S12. Dependence of the efficiency on D_{bot}, D_{top}, P, and L_{bot} when L_{top} = 8000 nm for the HE_{11} maximum in the GaInP/InP dual junction nanowire solar cell. For the separate variation of each parameter shown here, the other three parameters are kept at their optimized value (D_{bot} = 115 nm, D_{top} = 165 nm, P = 410 nm, and L_{bot} = 10200 nm give the optimized η = 38.0 %; see Table S1).

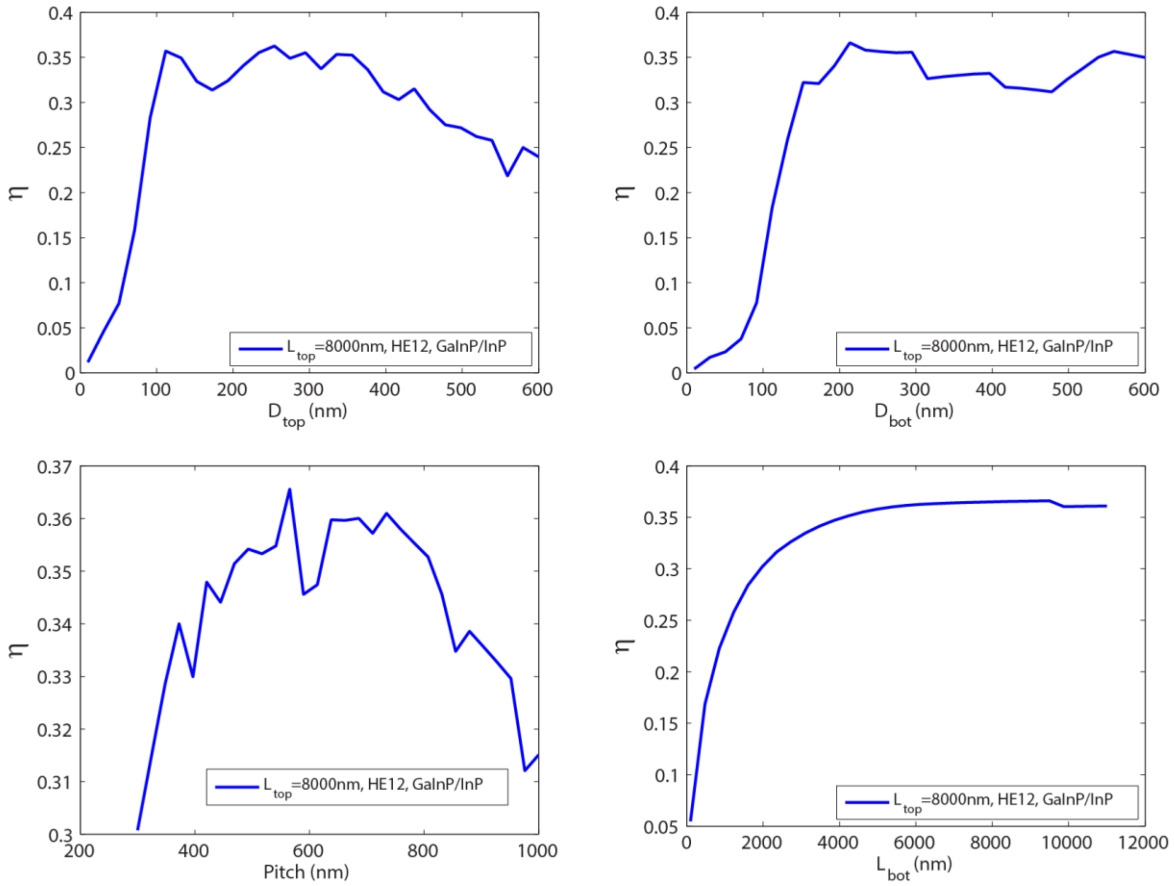

Figure S13. Dependence of the efficiency on D_{bot}, D_{top}, P, and L_{bot} when L_{top} = 8000 nm for the HE_{12} maximum in the GaInP/InP dual junction nanowire solar cell. For the separate variation of each parameter shown here, the other three parameters are kept at their optimized value (D_{bot} = 290 nm, D_{top} = 210 nm, P = 600 nm, and L_{bot} = 9600 nm give the optimized η = 36.6 %; see Table S1).

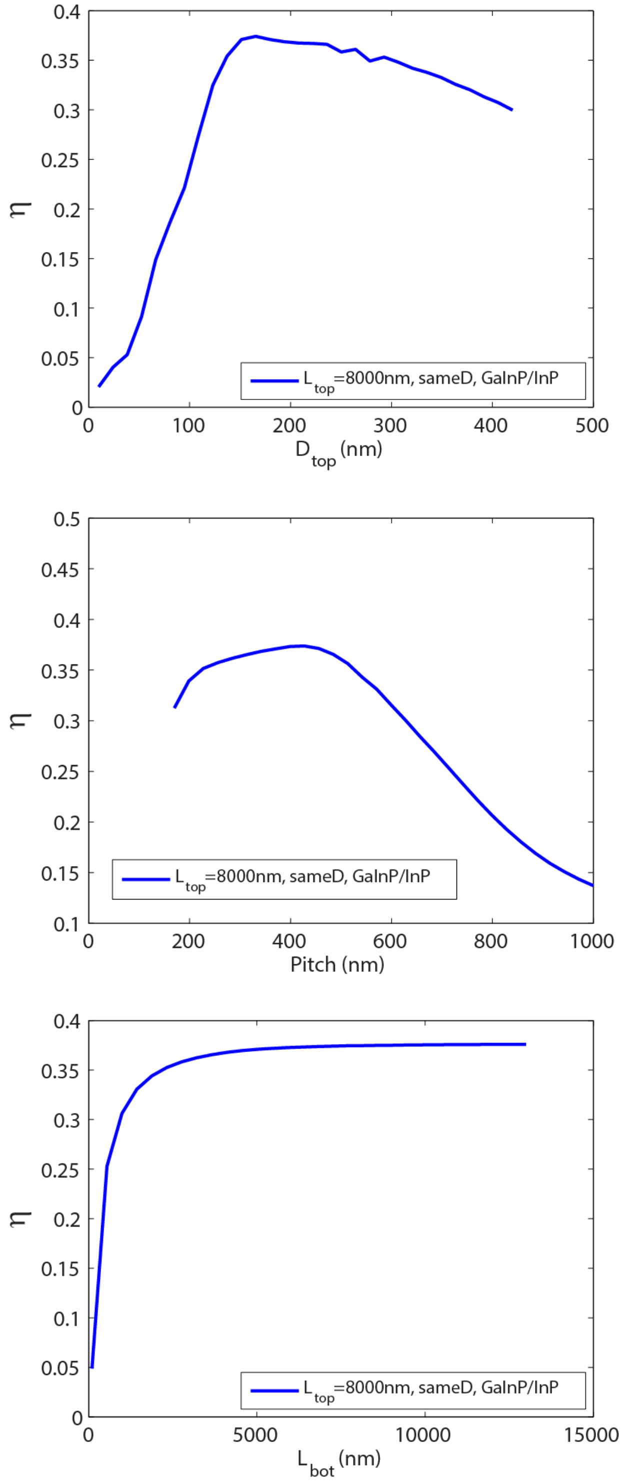

Figure S14. Dependence of the efficiency on $D_{bot} = D_{top}$, P, and L_{bot} when L_{top} = 8000 nm for the GaInP/InP dual junction nanowire solar cell. For the separate variation of each parameter shown here, the other three parameters are kept at their optimized value ($D_{bot} = D_{top}$ = 160 nm, P = 420 nm, and L_{bot} = 8800 nm give the optimized η = 37.5 %; see Table S1).

References

1 Devos, A. Detailed balance limit of the efficiency of tandem solar-cells. *J. Phys. D.* **13**, 839-846 (1980).

2 Anttu, N. Shockley-queisser detailed balance efficiency limit for nanowire solar cells. *ACS Photonics* **2**, 446-453 (2015).

3 Shockley, W. & Queisser, H. J. Detailed balance limit of efficiency of p-n junction solar cells. *J. Appl. Phys.* **32**, 510 (1961).

4 Brown, A. S. & Green, M. A. Impurity photovoltaic effect: Fundamental energy conversion efficiency limits. *J. Appl. Phys.* **92**, 1329-1336 (2002).

5 Sandhu, S., Yu, Z. & Fan, S. Detailed balance analysis and enhancement of open-circuit voltage in single-nanowire solar cells. *Nano Lett.* **14**, 1011-1015 (2014).

6 Sandhu, S., Yu, Z. & Fan, S. Detailed balance analysis of nanophotonic solar cells. *Opt. Express* **21**, 1209-1217 (2013).

7 Wang, S., Yan, X., Zhang, X., Li, J. & Ren, X. Axially connected nanowire core-shell p-n junctions: A composite structure for high-efficiency solar cells. *Nanoscale Res. Lett.* **10**, 22 (2015).

8 Yu, S. Q. & Witzigmann, B. A high efficiency dual-junction solar cell implemented as a nanowire array. *Opt. Express* **21**, A167-A172 (2013).

9 Anttu, N. & Xu, H. Q. Efficient light management in vertical nanowire arrays for photovoltaics. *Opt. Express* **21**, A558-A575 (2013).

10 Wang, X. F., Khan, M. R., Lundstrom, M. & Bermel, P. Performance-limiting factors for gaas-based single nanowire photovoltaics. *Opt. Express* **22**, A344-A358 (2014).

11 Létay, G. & Bett, A. Etaopt–a program for calculating limiting efficiency and optimum bandgap structure for multi-bandgap solar cells and tpv cells. *17th EC-PVSEC Munich, Paper VA1.25* **20** (2001).
